# Causes of admission, length of stay and outcomes for common kestrels in rehabilitation centres in the Czech Republic

**DOI:** 10.1038/s41598-021-96688-8

**Published:** 2021-08-26

**Authors:** Gabriela Lukesova, Eva Voslarova, Vladimir Vecerek, Marijana Vucinic

**Affiliations:** 1 Department of Animal Protection and Welfare and Veterinary Public Health, Faculty of Veterinary Hygiene and Ecology, University of Veterinary Sciences Brno, Brno, 61242 Czech Republic; 2 Department of Animal Hygiene, Faculty of Veterinary Medicine, University of Belgrade, 11000 Belgrade, Serbia

**Keywords:** Animal behaviour, Ecology

## Abstract

Rehabilitation centres help injured animals to recover and return back to the wild. This study aimed to analyse trends in intake and outcomes for the common kestrels (*Falco tinnunculus*) admitted into rehabilitation centres in the Czech Republic. From 2010 to 2019, a total of 12,923 kestrels were admitted to 34 rehabilitation centres with an increasing trend (rSp = 0.7697, *P* < 0.01) being found during the monitored period. Subadult kestrels (34.70%) and kestrels injured by power lines (26.57%) were most often admitted. Most kestrels in the rehabilitation centres died or had to be euthanized (81.66%), only 15.90% of the birds could be released back into the wild. The median length of stay in rehabilitation centres for kestrels that were subsequently released was 35 days. Considering survival rates, the most critical threat to kestrels was poisoning (100% of the cases resulted in death) but mortality of the kestrels admitted for most other reasons also exceeded 80%. Given the low success rate of the care of kestrels in rehabilitation centres and the relatively small proportion returned to the wild, it is essential to eliminate the causes leading to their admission, that is, to protect their natural habitats and to prevent unnecessary capture.

## Introduction

Raptors (*Falconiformes*) are a diverse group of birds, however, many of them are endangered species. Eighteen percent of raptors are threatened with extinction and 52% of raptors have declining global populations^1^. Threats to raptors come primarily from humans and include habitat loss, climate change, poisoning, collisions, electrocutions, deliberate trapping or shooting^2–5^. In result, raptors are often admitted to rehabilitation centres^6^. However, only a few studies focused on the intake and outcomes for raptors at rehabilitations centres so far. Furthermore, they usually have included data for multiple species while total number of birds and rehabilitation centres included in the analyses was very limited. Komnenou et al.^6^ analysed outcomes for 402 raptors from 19 species admitted to the Veterinary Medical Teaching Hospital in Greece during a 3-year period (1997–2000). Harris and Sleeman^7^ analysed medical records from 111 bald eagles and peregrine falcons admitted to the Wildlife Center of Virginia from 1993 to 2003. The release rate reported for raptors in those studies was not high, it ranged from 39%^7^ to 57%^6^. Given the limited data analysed, the question remains how effective rehabilitation of raptors is. Our study aimed to assess trends in reasons for admission and treatment outcomes for a single species, namely common kestrel. Data from all rehabilitation centres in the Czech Republic during a 10-year period were collected for analysis. The common kestrel was chosen because this species is widespread in the Czech Republic and in consequence it is the raptor species most frequently admitted into rehabilitation centres. The data on species admitted in low numbers would not allow such a thorough analysis. However, we believe that analysis of reasons of admission and outcomes for common kestrels reflect also the threats faced by other raptors and not only in the Czech Republic. We assessed the most common causes of admission of these animals to rehabilitation centres, time spent in rehabilitation centres and the outcomes of rehabilitation. Furthermore, we analysed mortality rates in birds admitted into rehabilitation centres for different reasons. Knowledge of these factors is important for the protection of animals especially in their natural habitat to mitigate impacts of anthropogenic and other factors, which negatively affect common kestrels in the wild.

The common kestrel (*Falco tinnunculus*) is a small raptor from the *Falconidae* family, widespread throughout Europe, large parts of Asia and Africa, and just as abundant in the Czech Republic^8^. It is also known as the European kestrel, Eurasian kestrel or Old World kestrel. According to the International Union for Conservation of Nature, the common kestrel is included into the LC (least concern) category, i.e. it is not directly threatened by extinction. However, the International Union for Conservation of Nature, in agreement with some studies, draws attention to the declining trend in the number of these birds^9^. The common kestrel, like many other animal species, is adversely affected by anthropogenic activity. This activity results in deaths and injuries that reduce the common kestrel's quality of life and thus its populations often decline^10^. Intensive agriculture also contributes to the decline of common kestrels^11^. In the past, it has been mainly due to the use of certain toxic substances, such as organochlorides and biphenyls^12,13^. In the case of poisoning in raptors, it is also important to mention lead poisoning, caused not only by the use of lead ammunition but by secondary poisoning as well^14^. As anthropogenic activity changes, its effects on kestrel populations may vary over time. For example, in the second half of the twentieth century Newton et al.^15^ described a gradual reduction in mortality of sparrow hawks and kestrels due to organochloride poisoning and gunshots and, conversely, an increase of mortality after colliding with traffic. Similarly, the risks to raptors may vary depending on the habitat they inhabit^16^. Raptors living in the forest are endangered by logging and other species are affected by agriculture^1^. In addition to poisoning, raptors are also frequent victims of collisions with power lines^17^.

Despite the negative effects of anthropogenic activity, the common kestrel is able to coexist with people to some extent and live in their immediate vicinity. However, living in human closeness changes their way of life^18^ and nesting close to humans can have other negative effects on populations^19^. For example, there is a possibility that kestrels that nest in cities for a long time have lower genetic variability than their natural relatives, due to the isolation of these populations^20^. Other problems leading to unsuccessful nesting and raising of the nestling include the collection of their eggs and other inappropriate human interventions^21^. The factors that lead to a reduction in the number of common kestrels have been examined by several authors with varying results. Forero et al.^22^ refuted the assumption that the lack of natural habitats and a higher number of competing raptors contributed to the decline in kestrel numbers. Undoubtedly, the common kestrel, like any wild species, is also threatened by other factors that are not caused by humans, such as certain diseases^23^.

In many countries, care for wild animals (usually that are injured by anthropogenic causes) is provided by rehabilitation centres. Their main goal is to cure these animals and release them back to their natural habitat. This effort involves a range of tasks from the first examination after admission to the rehabilitation centre and determination of the prognosis with regard to the animal's condition and welfare during treatment and up to rehabilitation, which increases the chances of its survival after returning to the wild.

## Results

**Figure 1:**
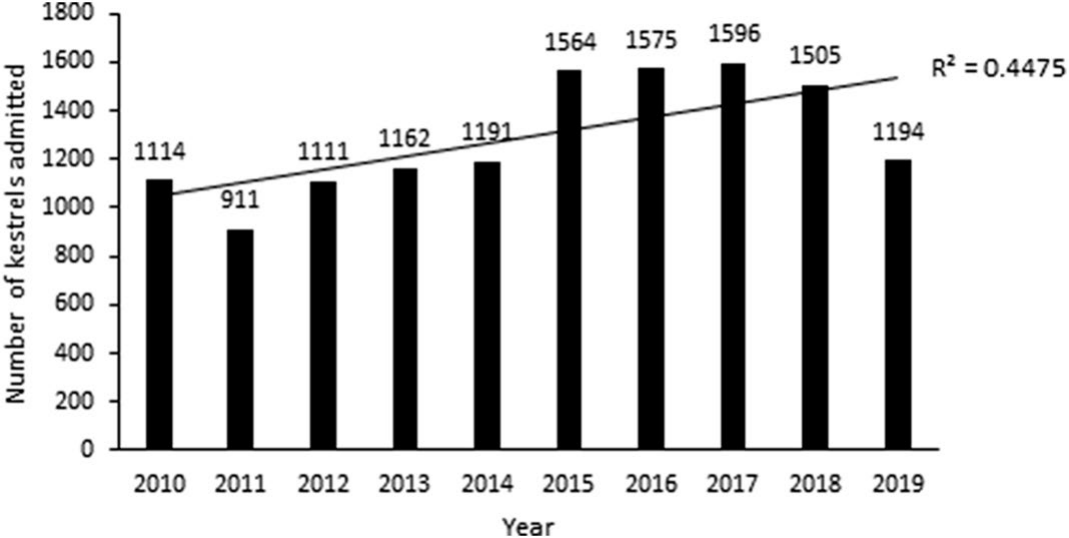
Number of common kestrels admitted to rehabilitation centres in the Czech Republic in the period from 2010 to 2019.

During the period from 2010 to 2019, a total of 12,923 common kestrels were admitted to 34 rehabilitation centres in the Czech Republic, with yearly intake numbers ranging from 911 to 1596 kestrels (Fig. 1). An increasing trend was found in the number of common kestrels admitted to the rehabilitation centres in the Czech Republic during the monitored period (rSp = 0.7697, *P* < 0.01).

The most common reasons for the admission of common kestrels to rehabilitation centres was admission of nestlings (34.70%), followed by admission due to power line injuries (26.57%), other injuries (18.13%), exhaustion, starvation (4.96%) and admission after collision with road traffic (4.57%) (Table 1). Other reasons accounted for less than 4% of admissions each.

**Table 1:** Number and percentage of the total number of common kestrels admitted to rehabilitation centres from 2010 to 2019 according to the reason for their admission.

Reason for admission	Admitted kestrels n = 12,923)	
	Number	%
Nestlings	4484	34.70^a^
Power line injury	3434	26.57^b^
Other injury	2343	18.13^c^
Exhaustion, starvation	641	4,96^d^
Road traffic	591	4.57^d^
Other	413	3.20^e^
Entrapment	220	1.70f.
Unnecessary capture	188	1.45^ fg^
Incubation	145	1.12^ h^^,i^
Feather damage	128	0.99^i^
Intrusion	126	0.98^i^
Injury by another animal	76	0.59^j^
Infection	63	0.49^j,k^
Rail traffic	26	0.20^ l^
Gunshot injury	25	0.19^ l^
Poisoning	20	0.15^ l^^,m^

^a-m^ The values with different superscript letters in a column are significantly different (*P* < 0.05).

Out of the 12,808 kestrels having their stay in the rehabilitation centre terminated in the monitored period, the majority of birds (81.66%) died or had to be euthanized in rehabilitation centres (Table 2). Significantly (*P* < 0.05) fewer birds were returned to the wild (15.90%). One hundred and fifteen kestrels were still being treated at the end of the monitored period thus they were not included in the outcome analysis.

**Table 2:** Number and percentage of the total number of common kestrels admitted to rehabilitation centres in the period from 2010 to 2019 according to the outcome.

Outcome	Kestrels (n = 12,808)	
	Number	%
Death	9920	77.45^a^
Release	2037	15.90^b^
Euthanasia	539	4.21^c^
Permanent captivity	173	1.35^d^
Unknown	134	1.05^e^
Escape	5	0.04f.

^a-f^ The values with different superscript letters in a column are significantly different (*P* < 0.05).

**Figure 2:**
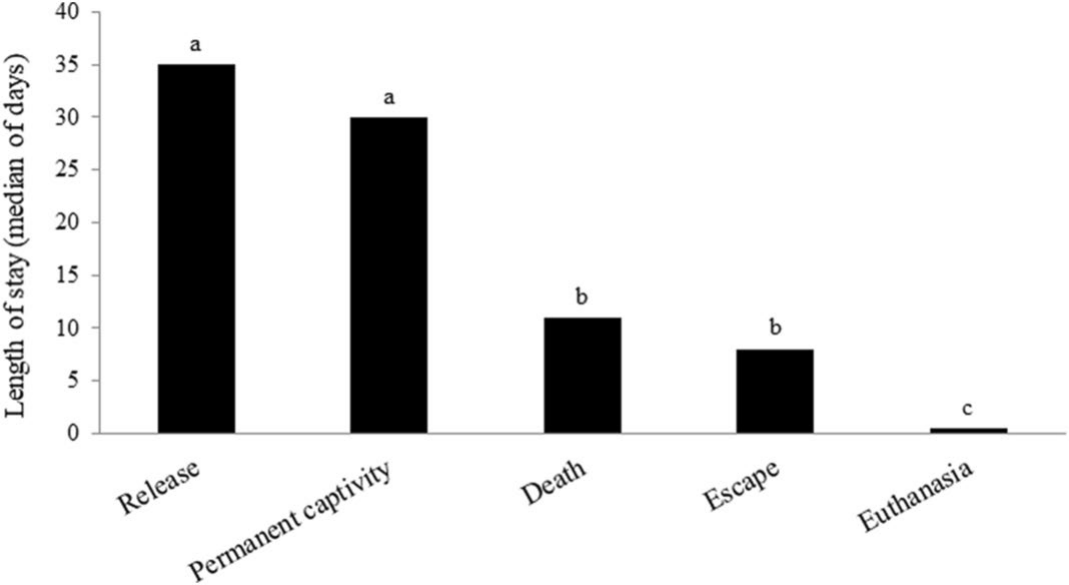
Comparison of the length of stay of common kestrels in rehabilitation centres depending on the outcome. ^a-c^The values in columns with different superscript letters are significantly different (*P* < 0.05).

The kestrels that were subsequently released back to the wild spent a median of 35 days in the rehabilitation centres (Fig. 2). The kestrels that were declared to be incapable of being released to the wild spent a comparable time (median 30 days) in the rehabilitation centres before they were transferred to another facility for permanent captivity. The decision to euthanize usually occurred within the first day (median 0.5 day, range 0.5—332 days) and deaths in treated birds occurred within 11 days (median 11 days, range 0.5—1,828 days). There were several cases of birds escaping within the first days after admission.

The results show that for almost all reasons for admission to rehabilitation centres, mortality was higher than 50% (Table 3). In poisoning, mortality was 100%, with none of the poisoned kestrels being saved. There was also a high mortality rate among birds admitted to rehabilitation centres for reasons other than injury or disease, such as unnecessary capture (77.13%) or feather damage (76.56%). The highest survival rates were found in nestlings and those hatched in rehabilitation centres, but still their mortality was high (64.59% and 44.83%, respectively).

**Table 3:** The number of kestrels that died or had to be euthanized depending on the reason for their admission to rehabilitation centres.

Reason for admission	Number of admitted birds	Number of deaths	Mortality rate (%)
Nestlings	4484	2896	64.59
Power line injury	3434	2900	84.45
Gunshot injury	25	17	68.00
Injury by another animal	76	66	86.84
Other injury	2,343	1971	84.12
Road traffic	591	528	89.34
Rail traffic	26	23	88.46
Poisoning	20	20	100.00
Infection	63	61	96.83
Feather damage	128	98	76.56
Exhaustion, starvation	641	534	83.31
Entrapment	220	163	74.09
Intrusion	126	110	87.30
Incubation	145	65	44.83
Unnecessary capture	188	145	77.13
Other	413	323	82.09

## Discussion

During the monitored period, an increasing trend was found in the number of admissions of common kestrels to rehabilitation centres in the Czech Republic. However, the question remains whether this is caused by the growing number of injured kestrels in the wild or by the increased public attention to injured animals. There is not enough information to assess this trend. Some authors also found an increasing trend in the number of animals being admitted to rehabilitation centres^24–26^, but some studies have instead found a decreasing trend^27,28^. We can assume that there are differences given the different periods and species observed as well as varying legal regulation of wildlife protection and the people's attitude towards care for disabled wild animals found in the wild.

The reasons for admitting common kestrels to rehabilitation centres in the Czech Republic were very diverse. Most frequently admitted were nestlings (34.70%) and birds injured by power lines (26.57%). Similarly, Molina et al.^4^ reported the admission of nestlings as the most common reason for admission (32%) in a rehabilitation centre in Spain in the period from 1995 to 2007. These nestlings may be brought unnecessarily, as kestrels can nest in the immediate vicinity of humans^29^ and thus arouse their attention or pity. However, these may also be weakened young during periods without plentiful prey, thus reducing the success of nesting and the number of raised nestlings^30^. Kestrels protect their nests^31^, but the presence of humans or noise is stressful and may result in the abandonment of the nests^32^. The negative consequences of noise and other factors related to birds nesting near human dwellings were described by e.g. Baudains and Lloys^33^, French et al.^34^ and Halfwerk et al.^35^.

Worldwide, collisions and electrocution by power lines are responsible for the death of many birds, especially raptors. The number of carcasses near power lines may not accurately reflect the number of birds killed by power lines^2^ because some of the carcasses can be taken away by scavengers^36^. However, a high mortality rate was confirmed by various studies^3,37,38^. The mortality rate can be so high that it affects a population in the area^39^. Over a ten-year period, Rodríguez et al.^5^ observed a raptor rehabilitation centre in Tenerife, where common kestrels predominated among the admitted raptors, and found that various injuries, including collisions with power lines, were the most common reason for admission (42.2%). The birds that were not killed immediately may be left burned and unable to fly. Power lines, including unsafe power poles, can cause serious injuries to which raptors are particularly susceptible because of specific morphological features (such as their large size and wingspan) and behavior (frequent perching or nesting on power poles)^40–42^. More research is needed on the effects of application of mitigation measures and solutions must be developed on a case-by-case basis^43,44^. Some pylons can cause higher mortality in birds than others, depending on, for example, their height and position^45^ or technical design^46^. Individual measures must also be considered from the point of view of specific species or groups of birds. Differences in perching behavior and possibility of electrocution cause differences in effectiveness of modifications. Insulation of crossarm braces seem to be effective even for the small raptors including kestrels^47^.

Out of the 12,808 common kestrels with known outcomes during the monitored period, 9,920 (77.45%) birds died and 539 (4.21%) birds had to be euthanized due to incurable conditions or diseases. A successful recovery leading to release of the birds back to the wild was uncommon; it was the outcome for only 2,037 (15.90%) birds. Interestingly, a considerably higher percentage of released raptors was reported in some other studies. In Jordan, between 2017–2018, 55.80% of raptors admitted to a rehabilitation centre were released^48^; in Greece, between 1997–2000, 56.90% of raptors admitted to a clinic were released^6^; in Spain, between 2003–2013, 57.57% of raptors were released^49^; and in Tenerife, between 1998–2007, 44.4% of raptors admitted to a rehabilitation centre were released^5^. In all studies, the total number of raptors was assessed without distinction between various species. Thus, it is not clear how many or if any common kestrels were included and what release rates for individual raptor species were. Furthermore, while the release rates were low for common kestrels in the general wildlife rehabilitation centres operating in the Czech Republic, specialized raptor centres might be more successful when providing treatment to raptors showing the advantage of specialization in rehabilitation centres.

Treatment options for raptors are constantly improving, yet knowledge of the individual requirements of a given species is still very important, along with performing autopsies and identifying the factors that lead to deaths^50^. Evaluation of blood parameters may be helpful for adjustment of treatment^51^. Prognosis may also be influenced by other factors during the kestrel's life that animal rehabilitation workers are unable to identify. Some studies have addressed the causes of death in raptors, but none focused on species-specific causes. Sara and Craig^52^, in a rehabilitation centre in Alabama between 1999–2000, reported higher mortality in adult raptors than in young birds. They explain higher mortality in adult birds by older raptors being less tolerant of human handling. Human handling is highly stressful for raptors. As a consequence of stress they may refuse to feed, which further reduces their chances of recovery, especially in the case of long-term captivity^53^. Correspondingly, higher mortality in mature raptors (mortality rate 77.13%) admitted to rehabilitation centres including those that were healthy at the time of admission (unnecessary capture) in comparison with nestlings (mortality rate 64.59%) was found in our study. Furthermore, Sara and Craig^52^ reported higher mortality in raptor species that are naturally active during the day. They suggest that raptors with diurnal activity are more frequent victims of shooting or road traffic. This may also apply to common kestrels that are diurnal hunters.

The length of stay in the rehabilitation centre differed according to the outcome. Statistically, the kestrels that died or had to be euthanized spent a significantly shorter time in rehabilitation centres than those that were treated successfully and subsequently released. The decision to euthanize was usually made on the day of admission to the rehabilitation centre (median 0.5 days). However, even among the birds that were considered curable and provided with treatment, many of them died within a few days (median 9 days). Molina et al.^54^ found the same length of stay in cases of euthanasia for all animals admitted to a rehabilitation centre in Catalonia. Euthanasia administered shortly after admission is the result of a rapid assessment of the animal's health and its chances of recovery and return to the wild. This rapid decision-making is very important in minimizing the individual's suffering during treatment, especially when treatment success and the animal's return to the wild are uncertain or unlikely^55^. The results of our study, in which only a very small percentage of birds were euthanized compared to the total number of kestrels that died in treatment, suggest that rehabilitation centres in the Czech Republic made greater efforts to rehabilitation kestrels even in the case of an uncertain prognosis. Corresponding to this is the longer time spent in the rehabilitation centre before death found in our study (median 9 days) than found by Molina et al.^54^ in Catalonia (2 days). It remains to be seen whether this approach is appropriate from the point of view of bird welfare. Efforts to rehabilitation animals with a poor prognosis increase their suffering, so it is necessary to consider the benefits of treatment with regard to their welfare, especially for wild animals^56^. The finding of unassisted death being more prevalent than euthanasia was reported also by other studies exploring various species of animals admitted to rehabilitation centres, e.g. reptiles, amphibians, mammals or birds^54^ or hedgehogs^27^. Kelly and Bland^28^ reported the number of individuals that died or were euthanized after unsuccessful treatment (more than 40%) as comparable to those euthanized upon admission to the rehabilitation centre (approximately 35%) in Eurasian sparrow hawks (*Accipiter nisus*) admitted to a rehabilitation centre in England from 2000 to 2004.

Our study revealed that common kestrels admitted to rehabilitation centres had a very high mortality rate overall, almost regardless of the reason for admission. However, some differences in the chance of survival depending on the cause of admission were found. Kestrels admitted due to poisoning seem to have a zero chance of survival, none was rescued during the monitored period. Lead poisoning in raptors is often the consequence of the use of lead ammunition by hunters. Lead bullets are prohibited to be used in waterfowl, but not in other game, and secondary poisoning can occur in kestrels via their prey. For eagles and other raptors, lead is most toxic when consumed, as opposed to lead bullets or shot simply lodging in muscle tissue. Also widespread use of rodenticides is responsible for secondary poisoning to non-target species including raptors. Naldo and Samour^57^ found a high sensitivity to poisoning in the *Falconidae* family, noting that falcons are more susceptible to ammonium chloride poisoning than, for example, lead poisoning^58^. In our study, very high mortality rates were also found in kestrels admitted due to infectious diseases (96.83%), road traffic injuries (89.34%) and power line injuries (84.45%). Molony et al.^55^ also pointed out that mechanical injuries to animals are the most severe and common reasons for euthanasia. Survival can also be affected by finders attempting to take care of the birds on their own^59^. According to the Czech legislation, the care for injured wild animals can be provided only by the competent persons, i.e. persons holding a certificate of competence. Unfortunately, not all people are aware that by law they are obliged to notify the authorized rehabilitation centre in case of finding an injured wild animal and that it is illegal to take such an animal home. However, the results show that not only birds that were admitted to rehabilitation centres due to injury or disease had been dying there. A high mortality rate (77.13%) was found also in birds that were captured and brought to the rehabilitation centre unnecessarily.

The results of the study show that the success rate of the care for common kestrels in rehabilitation centres in the Czech Republic during the monitored period was not particularly high. In order to strengthen the protection of kestrels, it is, therefore, essential to primarily focus on their protection in the wild and eliminate factors contributing to the various causes of death and injury. Such measures include the minimization of human interventions in nature, the protection of habitats and the elimination of other risk factors, in particular those related to anthropogenic activity, as they have a major impact on the mortality and morbidity of raptors^6^. As shown by results of our and many other studies, one of the biggest threats to avian species are electric power lines. To reduce risks, it is possible to alter the poles and lines so that they are less dangerous to raptors^60^. The protection of raptors would be improved by greater safety of electrical infrastructure^61^. It is also important to educate the public in order to reduce the number of birds caught unnecessarily or to prevent unprofessional treatment of birds provided by the enthusiastic but incompetent finders. To ensure the successful return of birds from rehabilitation centres to the wild, it is essential to release healthy individuals at suitable locations that increase their survival chances. The time spent in captivity does not seem to impair prey-capture abilities of predation in the common kestrel^62^ yet in many respects, it is useful to monitor survival rates of raptors released after rehabilitation^63^.

## Conclusion

National rehabilitation centre data provide valuable information on the causes of injuries in common kestrels and thus the threats posed by human-altered habitats. Rehabilitation centres generally play an important role in caring for injured, diseased or displaced animals in an effort to release them back to the wild, but the results of our study show that in kestrels, this care is not as successful as in other species. Only a very small portion of the admitted birds could return to the wild. It is therefore essential to focus on prevention, i.e. on factors leading to admission of kestrels to rehabilitation centres. Many kestrels were admitted to rehabilitation centres due to the consequences of anthropogenic activity, especially power line injuries. The technical solutions to this problem already exist, but it is necessary to apply them more widely and effectively. The admission of young kestrels could be often prevented by elimination of human interventions during nesting. Public education is crucial also to avoid unnecessary capture of birds and risks posed to birds by domestic animals, use of chemicals and nettings.

## Methods

The data on common kestrels admitted to rehabilitation centres in the Czech Republic were obtained from the records of the Ministry of the Environment as the central state administrative authority in environmental affairs. The subject of the analysis was the records collected from all rehabilitation centres falling under the National Network of Rehabilitation Centres of the Czech Republic from January 1, 2010 to December 31, 2019. The rehabilitation centres are obligated to provide each injured wild animal found with a complex care routine including first aid, veterinary treatment, therapy and rehabilitation. A handicapped animal is defined by Czech legislation as a wild animal that is temporarily or permanently unable to survive in the wild due to injury, illness or other factors^64^. The records kept by rehabilitation centres contain data on all common kestrels admitted to the rehabilitation centres in the Czech Republic including the reason and date of admission and the outcome for each individual bird. All rehabilitation centres included in the study were general wildlife rehabilitation centres as there are no specialized raptor centres in the Czech Republic. Operation of the rehabilitation centres within the National Network of Rehabilitation Centres of the Czech Republic including the record keeping duty is regulated by the law as they are all state-funded.

For the purposes of statistical evaluation, the kestrels were divided into groups by the year of admission to rehabilitation centres, the reasons for admission to rehabilitation centres and the outcomes. For common kestrels with known admission and discharge dates, the length of stay was also calculated and evaluated.

The categories of the reasons for the admission and outcomes groups correspond to the guidelines for the evidence of admitted animals in rehabilitation centres in the Czech Republic (Table 4). All young birds not yet fledged were included in the ‘Nestling ‘ category, other categories included mature birds only. The categories of outcomes are given in Table 5. Each bird was recorded only in one admission and one outcome group.

**Table 4:** Reasons for admission of common kestrels to the rehabilitation centres in the Czech Republic.

Reason for admission	Description	Example
Nestling	All young birds that have not reached the age for leaving their nest, not able to survive on their own	Abandoned, orphaned, injured hatchlings or nestlings
Power line injury	Injuries caused by the electrical charge from a power line	Kestrels lying near a power line or found with burn injuries (collisions and electrocutions)
Gunshot injury	Injuries caused by rifles, firearms	Gunshot wounds found in the wing etc
Injury by another animal	Injuries caused by a bite	Kestrels admitted after being seen to be attacked e.g. by a dog, suffering from bite wounds
Other injury	Other injuries, which were not caused by gunshot, power line or by a bite	Fractures, paralysis etc
Road traffic	Kestrels injured by road vehicles	Injured kestrels found on the side of the road
Rail traffic	Kestrels injured by rail cars	Injured kestrels found near the railway tracks
Poisoning	Kestrels showing signs of poisoning	Kestrels showing signs of poisoning (e.g. neurological signs after ingestion of carbofuran), no obvious injuries
Infection	Kestrels showing the symptoms of infection (including parasite infection)	Symptoms include diarrhea, ruffled feathers, sneezing etc
Feather damage	Inability to fly due to the feather damage	E.g. oil contamination
Exhaustion, starvation	Dehydration or underweight, no symptoms of infection	Kestrels showing signs of dehydration
Entrapment	Kestrels trapped in some way	Kestrels found tangled in nettings or in a chimney
Intrusion	Kestrels that invaded a building, namely when they could not get out	Kestrels found in a building
Incubation	Kestrels hatched in rehabilitation centres	Incubation of eggs collected from a damaged nest
Unnecessary capture	Kestrels (nestlings) brought into the rehabilitation for no apparent reason	Healthy kestrels showing no signs of injuries or diseases brought into the rehabilitation centre by a member of the public
Other	Reasons other than those described above	E.g. confiscation of illegally kept kestrels, capture of birds escaped from aviaries or falconers

**Table 5:** Outcomes for common kestrels in the rehabilitation centres in the Czech Republic.

Outcome	Description
Release	Kestrels that recovered and were released back to their natural habitat
Death	Kestrels that died in the rehabilitation centres (unassisted death)
Euthanasia	Kestrels euthanized for health reasons by a veterinarian
Permanent captivity	Kestrels that had to remain in captivity due to permanent disability
Escape	Kestrels that escaped during handling of birds or their treatment or transport in the open air
Unknown	Not specified

We calculated the length of stay of the kestrels in rehabilitation centres as the difference between the date of admission and the date when the stay of a kestrel in the rehabilitation centre was terminated. The kestrels for which the dates were not recorded and those that were still treated in the rehabilitation centre at the end of the monitored period were excluded from this analysis as it was not possible to determine their length of stay. For kestrels which were admitted on the same day as their record was terminated, the length of stay was set to 0.5 days. It is an estimate of the time needed to determine the diagnosis and prognosis (euthanasia, release after treatment, etc.). The median length of stay was calculated separately for each outcome group.

The data were evaluated using the statistical program UNISTAT 6.5 for Excel (Unistat Ltd., London, UK). In assessing the change in the number of recorded common kestrels, we used the Spearman coefficient to determine the coefficient of order correlation. A chi-square test with Yates correction within the methodology of 2 × 2 contingency tables was used for statistical evaluation of frequency differences within individual groups with numbers of individuals > 5. We used the Kruskal–Wallis ANOVA (and multiple comparisons for t-distributions) to statistically compare the length of stay among the individual categories of birds according to their outcome. In the tests used, the value of *P* < 0.05 was determined to be statistically significant.
